# LPC-transformer: a training strategy optimized framework for multiclass pneumonia medical image classification

**DOI:** 10.3389/fmed.2026.1960913

**Published:** 2026-09-22

**Authors:** Hongwei Chang, Tao Hu

**Affiliations:** 1 Shanxi Vocational University of Culture and Tourism, Institute of Digital Technology, Taiyuan, China; 2 School of Life Sciences and Technology, Xi'an Jiaotong University, Xi'an, China

**Keywords:** LMF hybrid loss, lung x-ray and CT imaging, polyReLU, scion optimizer, training strategy optimization

## Abstract

Lung imaging enables direct visualization of lesions and is vital for pneumonia diagnosis. However, current deep learning models show unsatisfactory classification accuracy and generalization for multi-class lung disease recognition, limiting their clinical decision-support value. To tackle this issue, we present LPC-Transformer, a framework optimized via improved training strategies with Swin Transformer as the backbone; performance gains are realized without deepening the network. We propose an LMF hybrid loss integrating Focal Loss and LDAMLoss to mitigate class imbalance. Furthermore, the SCION optimizer is adopted to stabilize training, and PolyReLU is used in MLP modules to model complex nonlinear features. Evaluated on CL-COVIDset containing 11 classes of lung CT images, our method is compared with mainstream benchmark networks. Experiments reveal that LPC-Transformer surpasses the baseline SwinT model substantially, with accuracy, recall, precision and F1-score improved by 4.2%, 4.4%, 2.0% and 3.8%, respectively. These results validate the effectiveness of the proposed training strategy optimization.

## 1 Introduction

Pneumonia is one of the most common and high-risk respiratory diseases with diverse clinical subtypes, each exhibiting highly similar lung lesion characteristics in medical imaging. Accurate identification and classification of different pneumonia types are critical for formulating targeted clinical treatment plans and reducing patient mortality. Medical imaging technologies (1), including X-radiation (x-ray), computed tomography (CT), and magnetic resonance imaging (MRI), have become the mainstream and intuitive diagnostic tools (2) for pneumonia screening and differential diagnosis in clinical practice. Compared with traditional invasive detection methods (3), imaging diagnosis features non-invasiveness, high efficiency and low cost. Therefore, extracting fine-grained lesion feature differences from medical images to realize accurate automatic classification of multiple pneumonia types has important clinical application value and research significance for intelligent auxiliary diagnosis.

In actual clinical diagnosis, the high similarity of imaging manifestations among different pneumonia subtypes brings great challenges to accurate discrimination. Different types of pneumonia only show subtle differences in lesion distribution, texture and edge features in imaging images. Traditional manual diagnosis relies entirely on the clinical experience of physicians, which is prone to misdiagnosis and missed diagnosis due to visual fatigue and subjective judgment errors. Meanwhile, conventional traditional image analysis methods mainly rely on manual designed feature operators to extract shallow visual features, which cannot effectively capture high-dimensional semantic features and subtle lesion differences of pneumonia images. Such methods are difficult to adapt to the high-precision differential diagnosis requirements of multi-category pneumonia, resulting in limited diagnostic accuracy and poor robustness.

With the rapid development of artificial intelligence technology, deep learning has been widely applied and achieved remarkable results in the field of medical image classification, effectively breaking through the technical bottlenecks of traditional diagnostic methods (4). Different from traditional manual feature engineering, deep learning methods can automatically mine hierarchical feature information from labeled medical image datasets through end-to-end training (5). Current research on deep learning-based pneumonia classification mainly focuses on three core optimization directions: designing innovative network architectures, constructing multi-structure hybrid neural networks (6), and embedding optimized functional modules into existing mature models to strengthen feature learning capabilities (7). A large number of advanced intelligent diagnosis models have been proposed, which greatly promote the intelligent development of pneumonia imaging diagnosis.

Current deep learning models for medical image classification are mainly divided into two mainstream architectures: Convolutional Neural Networks (CNNs) and Transformer architectures based on self-attention mechanism (8). As the classic representative of CNNs, ResNet relies on stacked convolution layers and residual connection structures to extract local spatial features of images. It has outstanding advantages in capturing texture, edge and detail features of lung lesions, with low computational complexity and stable training performance, and has long been the basic backbone of medical image classification tasks. However, restricted by the fixed receptive field of convolution operation, ResNet and other CNN-based models cannot effectively capture long-range global dependency relationships in high-resolution pneumonia images, resulting in insufficient mining of global semantic information of lesions and limited classification accuracy for complex multi-type pneumonia (9).

Vision Transformer (ViT) makes up for the inherent defects of CNNs in global feature learning (10). By dividing images into patch sequences and combining global self-attention mechanism, ViT can establish long-distance feature correlation of lesion areas, and cooperate with sliding window mechanism to balance local detail feature extraction and global semantic modeling capabilities. It shows unique advantages in fine-grained classification of medical images and has become a mainstream architecture in current pneumonia diagnosis research. In addition, emerging sequence modeling architectures have gradually been applied in medical imaging tasks. MedMamba, a selective state space sequence model, adopts depthwise convolution to extract local spatial features and uses Mamba layers to process long-range dependency information (11). It has higher computational efficiency than Transformer, but its feature discrimination ability for subtle pneumonia lesions is insufficient, leading to unsatisfactory overall classification performance.

To integrate the dual advantages of CNN's local feature extraction and Transformer's global modeling, hybrid neural network models have become a hot research direction in medical image classification. The typical HiFuse model adopts a dual-branch parallel feature extraction structure, which uses CNN branch to extract local detailed features of pneumonia lesions and Transformer branch to capture global semantic features (12). This model achieves adaptive weighted fusion of dual-branch feature maps through an attention mechanism, achieving an overall accuracy of 97.35% on the test set of the LIDC-IDRI dataset, with AUC values for the three types of nodules all exceeding 0.96. However, the parallel processing of dual branches introduces redundant computational power, and there may be conflicts in feature extraction between the two branches, which could even have a counterproductive effect on feature extraction. The complex structure is prone to overfitting.

In addition to network structure innovation, algorithm and component optimization have become important supplementary means to improve model performance in recent years, mainly including loss function optimization, activation function improvement and model interpretability enhancement. In terms of loss function optimization, hybrid loss functions have been proven to effectively solve the problem of medical data category imbalance and optimize classification accuracy (13). In terms of activation function improvement, learnable Piecewise Linear Activation Functions (PLUS) and Activation-Aware Feedforward Networks (AAMLPS) can effectively enhance the model's nonlinear fitting ability, achieving a 0.7% accuracy improvement in medical classification tasks (12). In terms of interpretability optimization, Grad-CAM visualization technology is widely embedded in Transformer backbone networks. It can intuitively display the model's lesion attention area, effectively improving the visual interpretability and diagnostic credibility of the model, and achieving a diagnostic accuracy of 0.97 for tuberculosis auxiliary diagnosis (14). The above studies fully prove that fine-grained optimization of training components and algorithms can effectively improve the comprehensive performance of medical image classification models (15).

Although existing network structure design and algorithm optimization methods have achieved good classification results in pneumonia diagnosis tasks, there are still obvious limitations in practical clinical application. Most existing advanced models rely on stacking network layers and expanding parameter scale to pursue higher classification accuracy, which leads to increasingly bloated model volume and sharply increased computational cost. Such models have high hardware deployment requirements and are difficult to apply in clinical terminal equipment. In addition, large-scale models rely on sufficient balanced datasets for training, and their generalization ability is severely limited when facing small-scale, unbalanced pneumonia medical datasets in actual clinical scenarios, which greatly restricts the practical application and popularization of intelligent pneumonia diagnosis models. In view of the above limitations, this paper focuses on training strategy optimization and proposes a high-generalization pneumonia classification model to make up for the deficiencies of existing research.

Aiming at the common defects of existing pneumonia classification models such as redundant parameters, high computational cost and poor adaptability to unbalanced medical datasets, this paper proposes a novel LPC-Transformer classification framework oriented to training strategy and component optimization. Different from the mainstream optimization idea of improving model performance by increasing network depth and expanding parameter scale, the core design idea of LPC-Transformer is to optimize the model training system in a fine-grained manner. By designing a hybrid loss function, introducing a new optimizer and improving the activation function, the model's feature learning ability, training stability and nonlinear fitting performance are comprehensively enhanced, so as to realize high-precision classification of multi-type pneumonia under the premise of maintaining Medium-scale model characteristics. The main innovative contributions of this paper are summarized in three aspects:

1. a Medium-scale LPC-Transformer framework is proposed, which achieves classification performance improvement through training strategy optimization rather than model structure expansion, effectively reducing computational overhead and improving model deployment practicability.
2. an LMF hybrid loss function is designed by integrating the advantages of Focal Loss and LDAMLoss, which effectively solves the problem of category imbalance in pneumonia medical datasets and improves the discrimination ability of the model for minority pneumonia samples.
3. the SCION optimizer and PolyReLU activation function are introduced to replace the traditional training components, which significantly enhances the model's training stability and complex nonlinear feature fitting capability.

## 2 Materials and methods

### 2.1 Datasets

This paper uses the Continual Learning of COVID-19 dataset for lung medical images. which is publicly available on the Kaggle platform at the following link: https://www.kaggle.com/datasets/mustai/continual-learning-of-covid19/data. This dataset includes over 5,011 medical images across 11 categories: normal lungs (16), Omicron/Delta, wild-type SARS-CoV-2, and other types of bacterial pneumonia in CT scans; as well as normal lungs, bacterial pneumonia, Middle East Respiratory Syndrome (MERS), Severe Acute Respiratory Syndrome (SARS),Omicron/Delta, and other types of viral pneumonia—totaling over 5,011 medical images across 11 categories (17). The dataset covers a diverse and extensive range of pneumonia types, providing the model with a wide variety of training samples, which enhances the model's performance in classifying pneumonia and improves its generalization ability. A selection of medical images from the dataset is shown in Figure 1.

**Figure 1:**
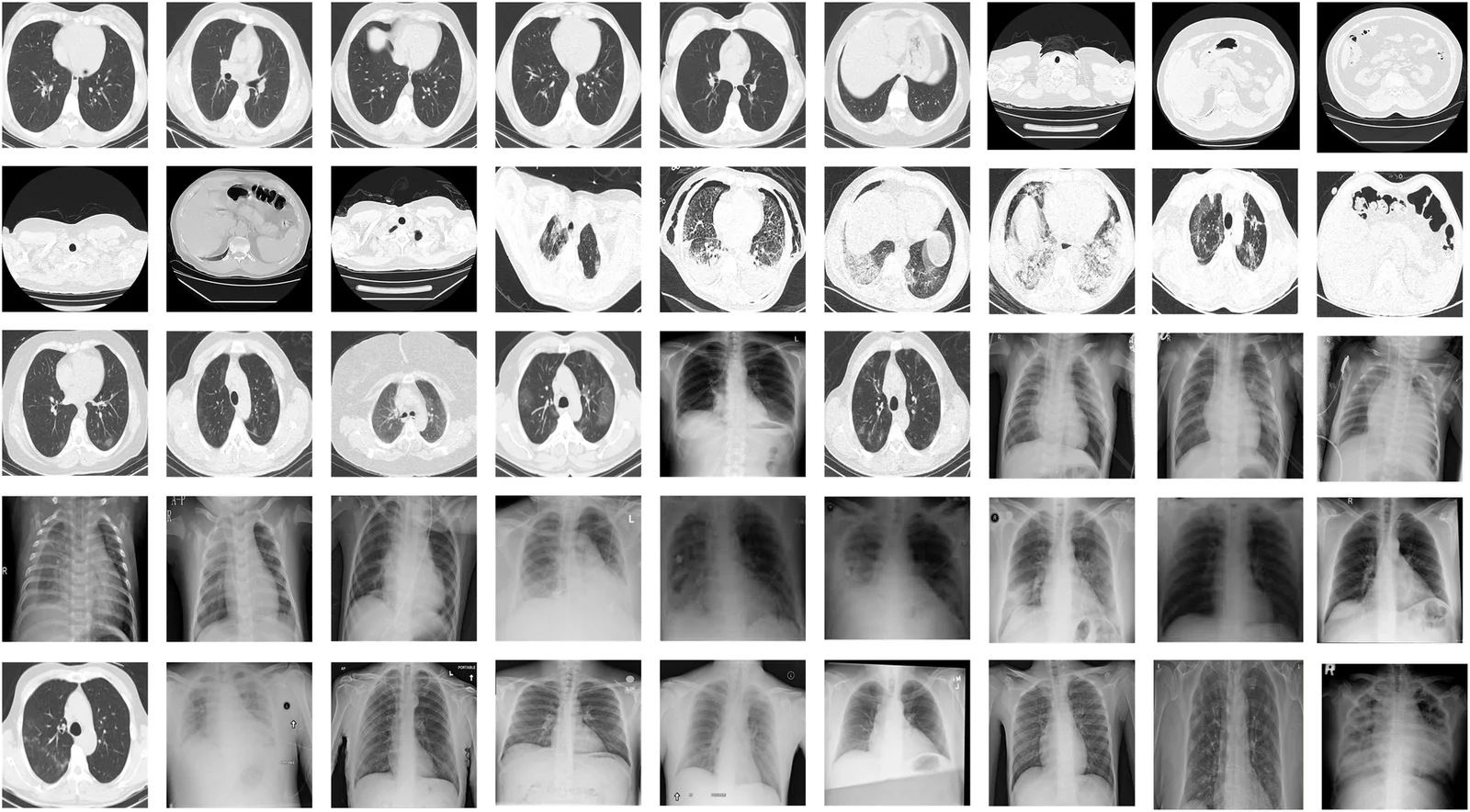
Examples of multimodal medical images in the dataset. The grid shows representative chest CT scans and x-ray images with varying pathological conditions and imaging characteristics.

The Continual Learning of COVID-19 dataset for lung medical images consists of three subsets: the training set, the validation set, and the test set. The samples are allocated to the training set, validation set, and test set in a 3:1:1 ratio (18). The training set is used to train the model and tune its parameters; the validation set is used to select the model and fine-tune its parameters to prevent overfitting; and the test set is used to evaluate the performance of the final model.

Specifically, out of the total 5,011 medical images, the test set comprises 1,072 samples. As illustrated in Figure 1, the distribution of categories within this test set exhibits significant class imbalance. For instance, the “CT Normal” category contains 285 samples, whereas “x-Ray SARS” has only 27 samples. This disparity, where the largest class is more than ten times the size of the smallest, presents a challenging evaluation scenario that rigorously tests the model's robustness against imbalanced data distributions. The number of samples for each category in the test set is shown in Figure 2.

**Figure 2:**
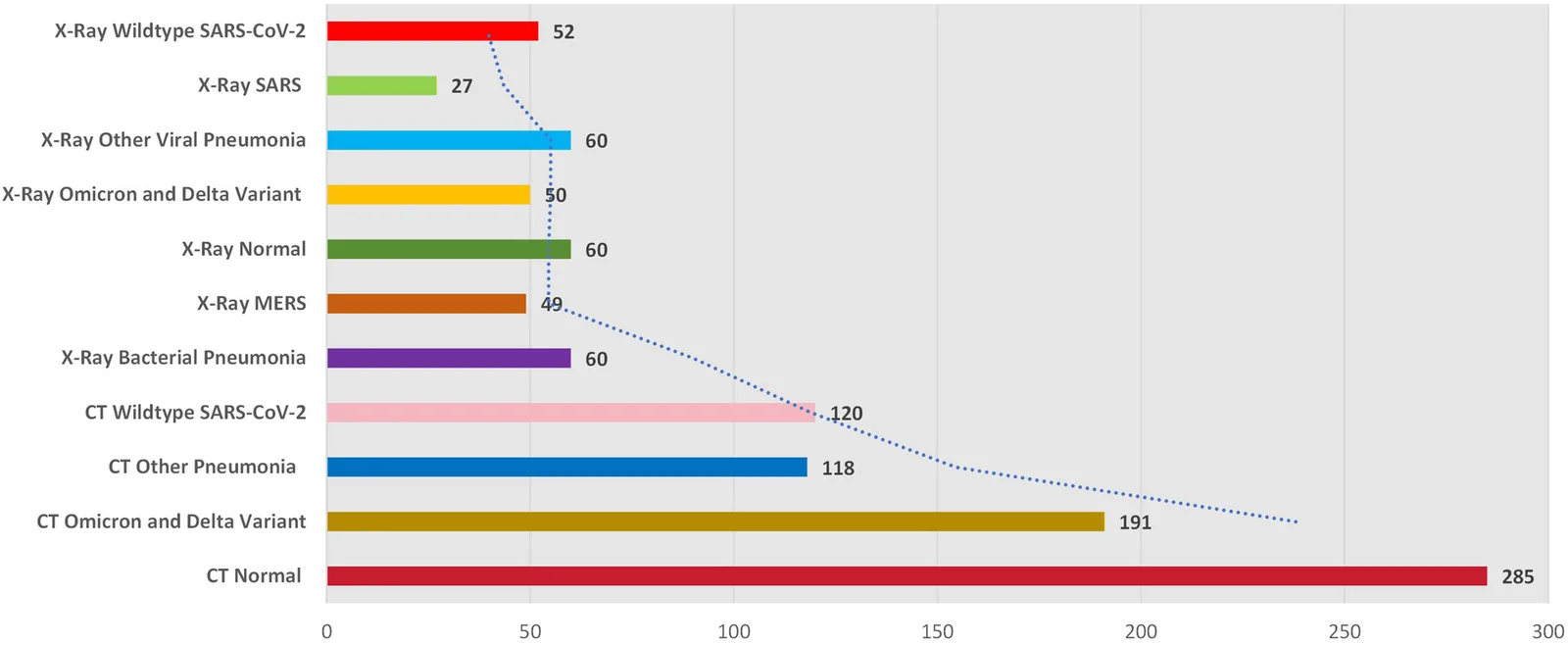
Distribution of sample counts across the 11 categories in the test set. The horizontal bars represent the number of images for each class, highlighting the significant class imbalance where “CT Normal” is the majority class (285 samples) and “x-Ray SARS” is the minority class (27 samples).

To further validate the generalization capability of the proposed model and demonstrate its robustness across heterogeneous data sources, a second publicly available dataset was introduced for supplementary evaluation. This dataset, the SARS-CoV-2 CT-Scan Dataset (19) is accessible on the Kaggle platform at https://www.kaggle.com/datasets/plameneduardo/sarscov2-ctscan-dataset/data. It comprises a total of 2,482 chest CT scans collected from real patients at hospitals in São Paulo, Brazil, of which 1,252 scans are positive for SARS-CoV-2 infection (COVID-19) and 1,230 scans belong to patients not infected by SARS-CoV-2. Unlike the first dataset, which encompasses a broad spectrum of pneumonia categories, this dataset is specifically designed for binary classification—distinguishing COVID-19-positive from COVID-19-negative cases—thereby providing a complementary and more focused evaluation scenario. By incorporating this independent dataset, the study not only strengthens the persuasiveness of the model's generalization ability but also provides empirical evidence that the proposed approach is not overly reliant on dataset-specific characteristics, thereby confirming its potential applicability in diverse real-world clinical environments.

### 2.2 Experimental environment and parameter settings

This experiment was implemented using Python within the PyTorch deep learning framework. The experimental platform was configured with a single P100 GPU with 16 GB of memory, running on a Windows 11 system.

After multiple rounds of experiments—both multi-round and single-round—the model achieved the best results with a single round of 200 training iterations. The input images consist of 224 × 224 × 1 lung medical image feature maps; the learning rate is set to lr=1e−3, and the batch size is batch_size = 32.

### 2.3 Model details

#### 2.3.1 Basic architecture

The Swin-Transformer (20) model, based on windowing, multi-level feature interaction, and a hierarchical attention mechanism, can simultaneously capture both global and local features in medical images and fuse them together. This model uses convolution operations to segment medical images into small patches and project them into high-dimensional vectors, thereby dividing the feature maps into windows. The feature map vectors undergo sequential processing across four stages. Except for the first stage, which performs linear embedding, the remaining stages first perform patch merging and downsampling on adjacent feature map windows, after which the feature map vectors are fed into a Swin-Transformer block for processing. This experiment uses 224 × 224 × 1 lung medical image feature maps (21); the complete processing workflow and the feature map vectors at each stage are shown in Figure 3.

**Figure 3:**
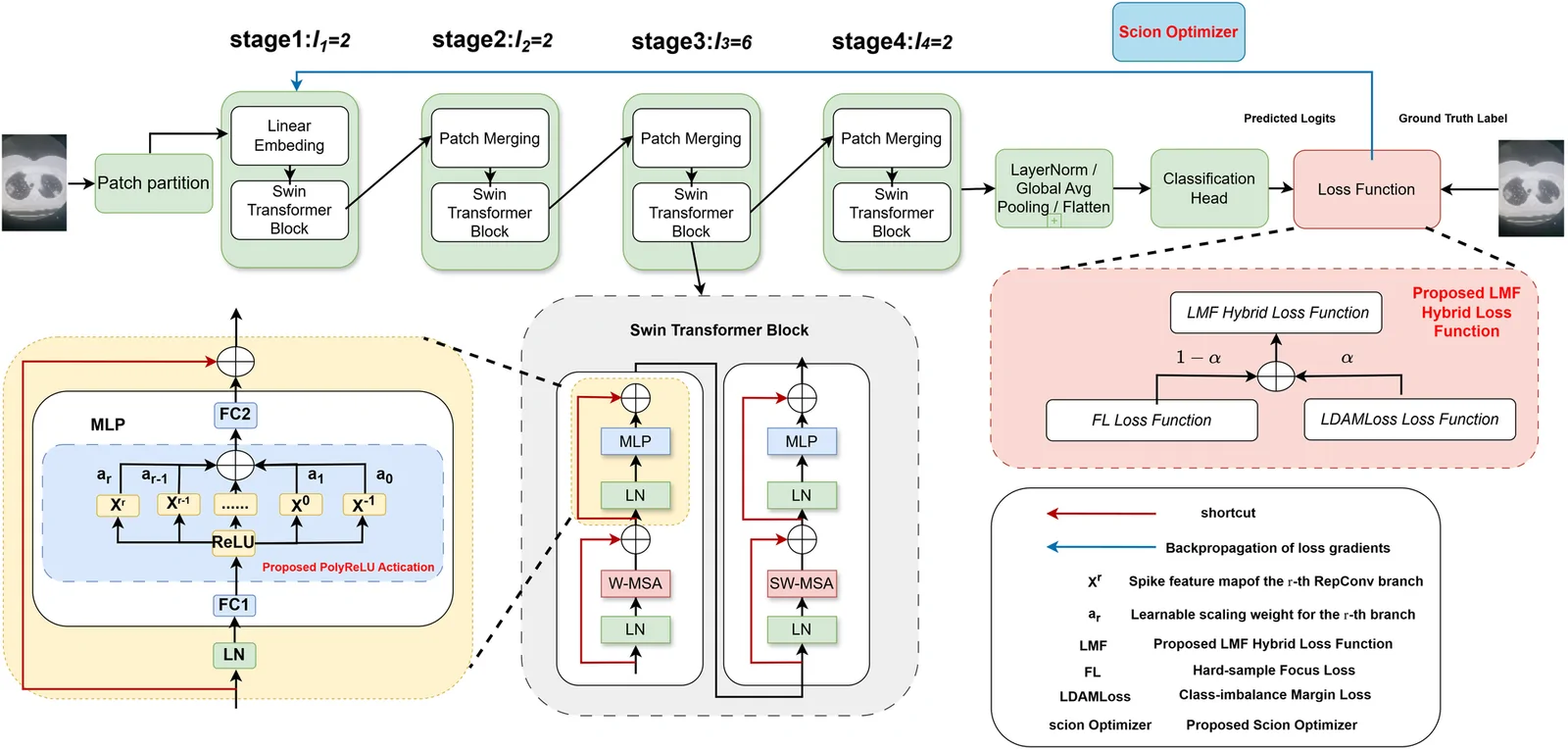
Architecture of the proposed Swin transformer model. Three core innovations are integrated: PolyReLU activation (bottom-left), weighted LMF hybrid loss (right-middle), and Scion Optimizer (top-right). The LMF loss combines FL and LDAMLoss to jointly address hard samples and class imbalance.

In each Swin Transformer Block module (22), the feature map vectors $z^{l-1}$ are normalized, and then the self-attention values for each window (Window-based Multi-head Self-Attention, W-MSA) are calculated, which are combined with the residual term $z^{l-1}$ to generate *ž*. This result is normalized again, passed through an MLP module for nonlinear processing, and the output $z^{l}$ is fed into the second block. The feature map is then re-segmented into new windows, and cross-window attention values are computed (Shifted Window-based Multi-head Self-Attention, SW-MSA). The attention (23) values are combined with the residual $z^{l}$ to produce *ž*. After undergoing normalization and processing through an MLP module, this stage is completed and outputs ${\hat{z}}_{l+1}$. In the tiny version of the Swin-Transformer, the number of blocks in each of the four stages is (2, 2, 6, 2) (24). Classification is performed after the output of the final stage. This process is illustrated in Figure 3.

#### 2.3.2 LMF hybrid loss function

Data missingness and non-standardization significantly impact the training results of neural networks. Loss functions have been proposed to address the issue of data imbalance. Focal Loss adjusts the weights of the loss function to make the model focus more on hard-to-classify samples, thereby improving classification performance. This loss function was initially used for object detection and has also performed well in classification problems. The formula is as shown in Equation 1:

$$FL(p_{t})=-a_{t}(1-p_{t}{)}^{\gamma }\log(p_{t})$$ (1)

Specifically, for $p_{t}$: when the true label is positive, $p_{t}=p$; when the true label is negative, $p_{t}=1-p$; and *p* is the probability predicted by the model.

LDAMLoss is also a loss function widely used in neural network models. By assigning different margin values to different classes, LDAMLoss enhances the separation of minority classes in the feature space. It is particularly suitable for addressing class imbalance issues. The formula is as shown in Equation 2:

$$LDAMLoss=\frac{1}{N}\overset{N}{\underset{i\;=\;1}{\sum }}\;w_{y_{i}}\log(p_{y_{i}}(x_{i}))$$ (2)

where N is the batch size, $x_{i}$ is the input feature of the i-th sample, $y_{i}$ is the true class index of the i-th sample, $p_{y_{i}}(x_{i})$ is the probability predicted by the model that the i-th sample belongs to its true class $y_{i}$, and $w_{y_{i}}$ is the weight corresponding to the true class $y_{i}$.

The LMF hybrid loss function combines the two loss functions above in a weighted manner, effectively leveraging both the Focal Loss function to handle hard-to-classify samples and the LDAMLoss to address class imbalance, resulting in a significant improvement in classification performance. The formula is as shown in Equation 3:

$$LMF=\alpha *LDAMLoss+(1-\alpha )*FL(p_{t})$$ (3)

where α is the weight of the LDAMLoss. Through extensive experimentation, it was found that setting α to 0.5 yields the best results.

#### 2.3.3 SCION optimizer

Swin-Transformer employs this approach for feature extraction; however, due to significant differences in network size across layers, issues such as unstable learning rates and suboptimal gradient updates arise. The optimizer is a critical component that controls the learning rate, updates gradients, and optimizes model parameters to minimize the loss function. SCION is an optimizer based on the Linear Minimization Oracle (LMO) that incorporates strategies such as hierarchical operational norms and hyperparameter transfer. This optimizer proactively adapts to the Swin-Transformer network architecture in advance, designs specific operational norms for each layer, and adapts to the varying network sizes across different layers of the Swin-Transformer through zero-order hyperparameter transfer. Ultimately, it generates parameter update methods for each layer. For example, given the weight matrix of a certain layer in the Swin-Transformer, $W\in R^{d_{out}\;\times d_{in}}$, the parameter update process of SCION is as follows:

1. Momentum accumulation. The momentum optimization process is achieved by combining the current gradient with historical momentum information, thereby accelerating convergence and reducing oscillations. The formula is as shown in Equation 4:
  $$d^{k}=0.9\cdot \nabla f(W^{k})+0.1\cdot d^{k-1}$$ (4)
  where: $\nabla f(W^{k})$ is the gradient at the weight matrix $W^{k}$, $d^{k-1}$ represents the momentum direction at the previous time step k-1, and 0.9 and 0.1 are coefficient parameters used to control the weight ratio between the current gradient and historical momentum.
2. LMO projection using spectral norm. The momentum direction is constrained by the spectral norm, and the direction is updated according to specific regularization conditions to improve training stability. The formula is as shown in Equation 5:
  $$lmo(d^{k})=\sqrt{\frac{d_{out}}{d_{in}}}\cdot UV^{⊤}$$ (5)

Where: $d_{out}$ represents the output dimension of the weight matrix, $d_{in}$ represents the input dimension of the weight matrix, and $UV^{⊤}$ is the matrix product, representing the low-rank approximation obtained from SVD.

(1) Parameter update. The model weights are adjusted using the projected momentum direction and the learning rate. The formula is as shown in Equation 6:
  $$W^{k\;+\;1}=W^{k}+\gamma_{k}\cdot lmo(d^{k})$$ (6)

where $W^{k\;+\;1}$ denotes the updated weight matrix, $W^{k}$ denotes the weight matrix at the current time *step k*, and $\gamma_{k}$ is the learning rate parameter.

#### 2.3.4 PolyReLU activation function

Activation functions are key components of neural networks. By mapping linear inputs to a nonlinear space, they enable neural networks to approximate and simulate functions of arbitrary complexity. In the Swin-Transformer model, the four-stage features require different nonlinear transformations. The PolyReLU activation function (25), with its independent multi-branch transformations of varying powers, better meets the Swin-Transformer's requirements for complex linear transformations. The formula is as shown in Equation 7.

$$PolyReLU(x)=\overset{r}{\underset{i\;=\;0}{\sum }}\;a_{i}ReLU^{i}(x)$$ (7)

where $ReLU^{i}(x)=max\{x,0{\}}^{i}$.

After passing through the fully connected layer FC1, PolyReLU increases the dimension from C to 4C, resulting in a feature vector of shape [B, H, W, 4C]. This vector first undergoes a ReLU nonlinear transformation, followed by independent multi-branch transformations of different powers. The feature vectors from different power levels are then connected via residual connections. The process is illustrated in Figure 3.

#### 2.3.5 Evaluation metrics

This experiment involved the classification of 11 categories of pulmonary medical images. We conducted ablation experiments and comparative experiments on neural network models and activation functions, using an 11 × 11 confusion matrix for evaluation. In this matrix, each column represents the true labels of the samples, each row represents the predicted labels of the samples, and the diagonal of the matrix indicates the number of instances where the predicted label matches the true label. In the 11-class lung medical image classification experiment, taking the “CT Normal” class as an example: a True Positive (*TP*) (26) refers to a sample predicted to belong to the “CT Normal” class and that actually does belong to it; a True Negative (*TN*) refers to a sample predicted not to belong to the “CT Normal” class and that actually does not belong to it; a false positive (*FP*) refers to a sample whose actual class is not “Normal Lung in a CT Setting,” but which the model predicts to belong to that class; a false negative (*FN*) refers to a sample that belongs to the “Normal Lung in a CT Setting” class, but which the model predicts does not belong to that class. Evaluation is performed using recall, precision, and F1 score based on the samples (27).

Accuracy refers to the proportion of samples correctly predicted as true out of the total number of samples. The formula is as shown in Equation 8:

$$\mathrm{Accuracy}=\frac{TP+TN}{TP+TN+FN+FP}$$ (8)

Recall refers to the proportion of samples that are actually positive that were correctly predicted. The formula is as shown in Equation 9:

$$\mathrm{Recall}=\frac{TP}{TP+FN}$$ (9)

Precision refers to the proportion of samples that are actually positive among those predicted to be positive by the model. The formula is as shown in Equation 10:

$$\mathrm{Precision}=\frac{TP}{TP+FP}$$ (10)

The F1 score is a metric that balances precision and recall, taking both factors into account. The formula is as shown in Equation 11:

$$F1\_score=2\times \frac{Precision\;\times Recall}{Precision+Recall}$$ (11)

## 3 Results

### 3.1 Ablation experiments and analysis

To validate the effectiveness of the proposed method, this study designed experiments that progressively introduced different improvements and systematically evaluated the contributions of each module through ablation experiments. Based on the ablation results on the test set, the gradual addition of the LMF loss function, the PolyReLU activation function, and the SCION optimizer led to significant improvements in model performance. The experimental metrics are shown in Table 1. The accuracy of swinT is 0.8218; after sequentially adding the LMF, it improved to 0.8228; after adding SCION, it reached 0.847; and finally, after adding PolyReLU, it reached a peak of 0.8638. The F1 score simultaneously increased from 0.8105 to 0.8487, validating the cumulative effect of component stacking on model optimization. The LMF loss function accelerates model convergence by improving gradient computation; the SCION module enhances feature interaction capabilities; and PolyReLU optimizes the model's expressive power through nonlinear activation. Experimental data show that the SCION and PolyReLU modules contribute the most significant improvements to model performance.

**Table 1:** Ablation experiments.

Method				Metrics			
swinT	LMF	PolyReLU	SCION	Accuracy (%)	Recall (%)	Precision (%)	F1 score (%)
✓	–	–	–	82.18	80.03	83.59	81.05
✓	✓	–	–	82.28	80.13	83.55	81.20
✓	✓	✓	–	84.70	82.25	83.92	82.85
✓	✓	✓	✓	**86**.**38**	**84**.**41**	**85**.**66**	**84**.**87**

The experiments are conducted by progressively integrating modules (LMF, PolyReLU, SCION) into the SwinT baseline. Bold values indicate the best performance achieved by the complete model.

The activation function significantly improves model performance, and comparative experiments were conducted using different activation functions. As shown in Table 2, PolyReLU achieved the best results on the test set with an accuracy of 86.38% and an F1 score of 84.87%, outperforming the second-best activation function, ReLU, by 0.37% and 0.96%, respectively. Its balance between Recall (84.41%) and Precision (85.66%) is also notable, demonstrating strong performance in distinguishing both positive and negative samples.

**Table 2:** Performance comparison of different activation functions.

Activation Function/Metric	Accuracy (%)	Recall (%)	Precision (%)	F1 score (%)
Gelu	84.70	82.25	83.92	82.85
Relu	86.01	83.46	84.66	83.91
SwiGLU	84.51	81.89	84.00	82.59
polyNorm	83.68	79.90	82.56	80.78
PolyReLU	**86**.**38**	**84**.**41**	**85**.**66**	**84**.**87**

We evaluate the impact of different activation functions on model performance. Bold values indicate that PolyReLU achieves the best results across all metrics compared to other candidates.

### 3.2 Comparative experiments and analysis

To fully validate the effectiveness of the LPC-Transformer method proposed in this paper, we selected MobileNetV2 (28), EfficientNet_b0 (29), ViT (30), ResNet50 (31), ConvNeXt (32), EfficientViT, HiFuse (33), and InternImage-B, to conduct comparative training experiments. The training accuracy curve is shown in Figure 4A. Throughout the full 200-iteration training period, the accuracy of the proposed model (red curve, LPC-Transformer) rose steadily and consistently. It achieved a rapid ascent in the early stages, reaching over 86% by the 40th epoch, and maintained a stable high-level performance thereafter. The final training accuracy reached approximately 87.9%, achieving the best performance among all compared models. This demonstrates that our model possesses superior fine-grained medical image feature extraction capabilities and robust training convergence characteristics.

**Figure 4:**
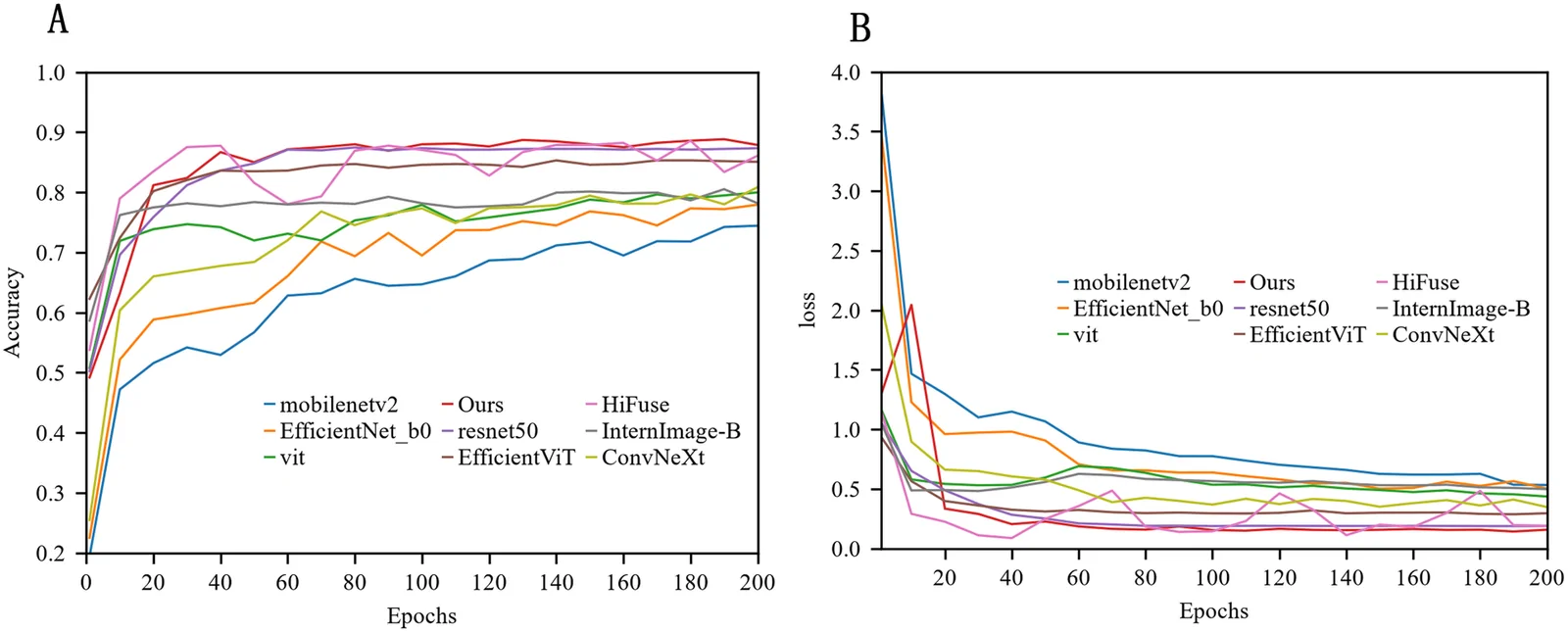
This figure shows the data variation curves obtained from 200 validation rounds on the validation set for different models. **(A)** Comparison of validation set accuracy among neural networks; **(B)** comparison of validation set loss function values among neural networks.

HiFuse (pink curve) showed strong competitiveness; although it experienced some fluctuations in the middle stages (dropping to around 81% at epoch 120), it recovered well in the later stages, eventually stabilizing near 86.1%, with overall performance second only to LPC-Transformer. ResNet50 (purple curve), as a classic convolutional baseline network, showed a relatively rapid increase in accuracy in the early stages and maintained a very stable convergence trend in the later stages, with the final accuracy maintaining around 87.4%, closely following our model. EfficientViT (brown curve) converged to an accuracy of approximately 85.1%, performing better than InternImage-B (gray curve, final accuracy roughly 78.2%). ConvNeXt (yellow-green curve) exhibited a generally stable upward trend, with its final accuracy stabilizing at 80.9%. ViT (green curve) is a pure Transformer baseline model, with a final convergence accuracy of approximately 80.0%, its feature extraction capabilities are weaker than the hybrid architecture models. EfficientNet_b0 (orange curve) showed slow accuracy improvement in the early training phase, with a moderate overall convergence rate, and a final accuracy of roughly 78.0%. The lightweight convolutional network MobileNetV2 (blue curve) converged the slowest; its accuracy improvement lagged behind the other models throughout the training process, with a final convergence accuracy of only 74.5%. It exhibits significant shortcomings in its ability to classify complex medical lesion images and cannot meet the requirements for high-precision diagnosis.

The loss function curves further demonstrate the training stability and generalization capabilities of each model, as shown in Figure 4B. The loss value of the LPC-Transformer (red curve) proposed in this paper decreased rapidly and significantly during the first 40 iterations—dropping from an initial value to below 0.5—and subsequently remained within the lowest loss range among all models throughout the rest of the training process. The curve is smooth with no sharp fluctuations, effectively suppressing overfitting in the later stages of training and achieving the best generalization performance. HiFuse (pink curve) exhibits a rapid rate of loss reduction in the early stages and maintains a very low loss value comparable to ours in the later stages, though with slightly more volatility. ResNet50 (purple curve), EfficientViT (brown curve), ConvNeXt (yellow-green curve), and InternImage-B (gray curve)—these four model types exhibit a mid-range level of loss convergence, gradually leveling off after approximately 60 iterations with only minor fluctuations, demonstrating acceptable training stability. ViT (green curve) maintains a loss higher than that of the top-performing models throughout the entire process, resulting in limited fitting performance. EfficientNet_b0 (orange curve) exhibited a slower rate of loss reduction compared to the top tier, with loss values remaining relatively higher. MobileNetV2 (blue curve) consistently had the highest loss values among all models and converged at the slowest rate, which corresponds to its accuracy curve performance, indicating that this lightweight model struggles to adequately capture the complex lesion features in medical images. Based on the two core metrics—accuracy and loss—over 200 training rounds, the LPC-Transformer proposed in this paper achieves the dual advantages of the highest convergence accuracy and the lowest steady-state loss, fully demonstrating that this model possesses both strong lesion feature extraction capabilities and excellent dataset generalization performance in medical image classification tasks.

To quantitatively assess the overall classification performance of the LPC-Transformer proposed in this paper, we conducted a comprehensive evaluation of various mainstream baseline models on a unified test set. The results for the four core evaluation metrics—Accuracy, Recall, Precision, and F1 Score—are shown in Table 3 and the bar chart in Figure 5.

**Table 3:** Quantitative classification metrics of mainstream state-of-the-art models and the proposed method.

Model/Metric	Accuracy (%)	Recall (%)	Precision (%)	F1 Score (%)
MobileNetv2_100	72.11	68.42	72.42	68.54
EfficientNet_b0	78.17	77.55	79.77	77.99
Vit_base_patch16_224	79.94	76.76	79.30	77.49
ConvNeXt V2	79.38	78.25	77.09	77.37
InternImage-B	76.68	80.48	76.44	77.09
EfficientViT	83.58	76.72	77.21	75.31
HiFuse	84.61	85.02	84.61	84.55
ResNet50	85.85	84.30	84.47	84.20
**Ours**	**86**.**38**	**84**.**41**	**85**.**66**	**84**.**87**

Comparison of the proposed method with nine mainstream state-of-the-art models. Bold values indicate that our method achieves the best performance across all evaluation metrics.

**Figure 5:**
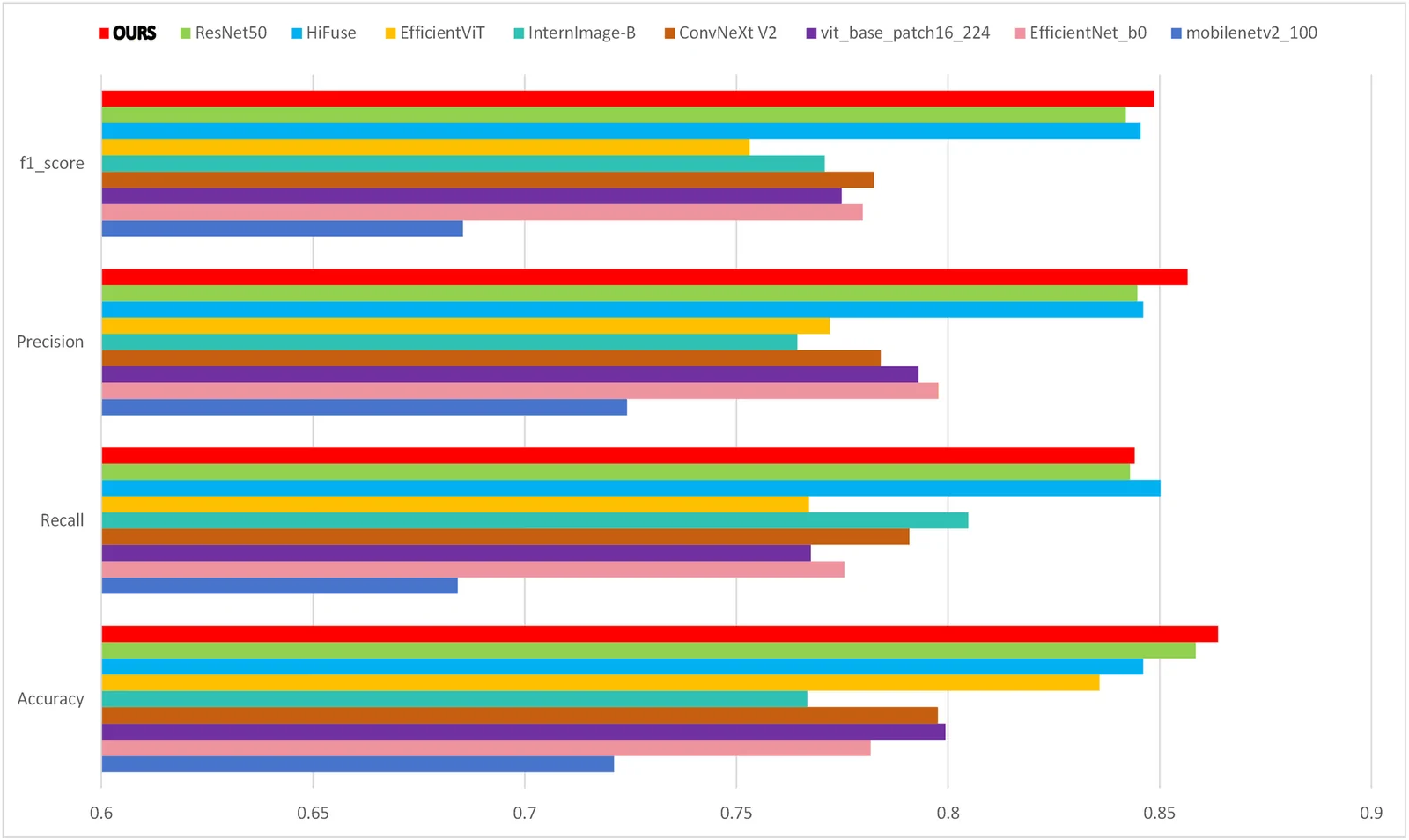
Comparison of the classification performance of nine models across four key metrics. The proposed LPC-Transformer (red bars) outperforms all competing models in Accuracy, Precision, and F1-score, and ranks second in Recall, demonstrating its strong overall performance.

The LPC-Transformer proposed in this paper achieved the best results across all models in the three core metrics—precision, recall, and F1 score—and outperformed all comparison models in overall performance. On the test set, it achieved a precision of 86.38%, a recall of 85.66%, an F1 score of 84.87%, and a recall rate of 84.41%, demonstrating balanced performance across all four metrics with no obvious weaknesses; The classic convolutional network ResNet50 ranked second in overall performance, with a precision of 85.85%, a recall of 84.47%, and an F1 score of 84.20%; only its recall was slightly lower than that of HiFuse; HiFuse ranked third, with a recall rate of 85.02%—the highest among all models—demonstrating greater sensitivity in identifying positive lesion samples in medical images. Its accuracy was 84.61%, precision was 84.61%, and F1 score was 84.55%, with overall metrics slightly inferior to those of ResNet50 and the model presented in this paper. The three hybrid and pure Transformer models—EfficientViT, ConvNeXt V2, and Vit_base_patch16_224—performed in the middle range: EfficientViT achieved an accuracy of 83.58%, but its recall, precision, and F1 score were significantly imbalanced, with an F1 score of only 75.31%, indicating poor balance in class identification; ConvNeXt V2 achieved a precision of 79.38%, with values for all metrics being relatively close; ViT_base_patch16_224 achieved a precision of 79.94%. The pure Transformer architecture lacks the ability to extract local features via convolutions, resulting in an overall performance that is weaker than that of convolutional-fusion models. EfficientNet_b0 and InternImage-B delivered average overall performance. EfficientNet_b0 achieved a precision of 78.17%, with relatively small variations across metrics; InternImage-B, with a recall of 80.48%, demonstrated some sample recognition capability, but its precision was only 76.44%, indicating a significant bias in positive and negative sample classification. The lightweight convolutional model MobileNetv2_100 ranked last in all four metrics, with an accuracy of only 72.11% and recall, precision, and F1 scores of 68.42%, 72.42%, and 68.54%, respectively. Its feature extraction capability is insufficient, making it difficult to adapt to medical image classification tasks with complex details and high category discrimination difficulty.

To further investigate the impact of the proposed hybrid loss function (LMF) on individual category performance, we computed the Precision, Recall, and F1 Score for each of the 11 categories under both the baseline configuration (standard cross-entropy loss) and the LMF-augmented configuration. The detailed per-category results are presented in Table 4. Among the majority classes with larger sample sizes, the introduction of LMF resulted in varying degrees of decrease: CT Normal (285 samples) exhibited reductions of 2.05% in Precision, 3.31% in Recall, and 2.60% in F1 Score; CT Omicron and Delta Variant (191 samples) showed a decrease of 1.54% in Precision, though its Recall slightly increased by 0.61%, resulting in a marginal F1 Score reduction of only 0.13%; CT Other Pneumonia (118 samples) and CT Wildtype SARS-CoV-2 (120 samples) experienced even smaller declines, with F1 Score reductions of 0.64% and an increase of 0.08%, respectively, indicating that the impact on these two categories was negligible. In contrast, the minority classes demonstrated consistent and notable improvements across all three metrics. x-Ray Bacterial Pneumonia (60 samples), x-Ray Normal (60 samples), and x-Ray Other Viral Pneumonia (60 samples) each gained modest but meaningful improvements in F1 Score (1.48%, 1.91%, and 2.17%, respectively). More importantly, the categories with fewer samples exhibited progressively larger gains: x-Ray Wildtype SARS-CoV-2 (52 samples) achieved an F1 improvement of 1.95%; x-Ray Omicron and Delta Variant (50 samples) gained 3.78%, with Recall increasing by 4.27%; x-Ray MERS (49 samples) achieved an F1 improvement of 3.09%, with Precision and Recall increasing by 2.99% and 3.18%, respectively. Most notably, x-Ray SARS—the smallest class with only 27 samples—received the most substantial boost, with Precision increasing by 7.95%, Recall by 8.46%, and F1 Score by 8.25%, representing the largest per-category improvement among all 11 categories. This inverse relationship between sample size and performance gain clearly illustrates the rebalancing effect of the hybrid loss function: by assigning higher penalization weights to underrepresented classes during training, LMF effectively redirects the model's learning focus toward the minority categories that are most vulnerable to being overlooked under standard loss functions. Although this mechanism incurs a performance trade-off on some majority classes (the largest decline being 2.60% in F1 Score for CT Normal), the net effect is a more equitable and robust classifier that achieves significantly improved recognition capability for rare but clinically critical pneumonia types—such as SARS and MERS—without sacrificing overall classification accuracy. These results provide compelling evidence that the proposed hybrid loss function plays a meaningful role in mitigating the adverse effects of class imbalance in multi-category medical image classification.

**Table 4:** Per-category performance comparison under the baseline cross-entropy loss and the proposed hybrid loss function (LMF).

Category Name	n	Precisi-on (%)	Recall (%)	F1 (%)	+LMF-Precision (%)	+LMF-Recall (%)	+LMF-F1 (%)	*Δ* Preci-sion (%)	*Δ* Recall (%)	*Δ* F1 (%)
CT Normal	285	77.88	91.26	84.04	75.83	87.95	81.44	−2.05	−3.31	−2.6
CT Omicron and Delta Variant	191	90.22	65.05	75.59	88.68	65.66	75.46	−1.54	0.61	−0.13
CT Other Pneumonia	118	90.87	89.64	90.25	90.3	88.94	89.61	−0.57	−0.7	−0.64
CT Wildtype SARS-CoV-2	120	95.96	78.67	86.46	95.35	79.22	86.54	−0.61	0.55	0.08
x-Ray Bacterial Pneumonia	60	74.67	82.58	78.43	76.54	83.59	79.91	1.87	1.01	1.48
x-Ray MERS	49	88.71	83.23	85.88	91.7	86.41	88.97	2.99	3.18	3.09
x-Ray Normal	60	87.93	84.16	86	89.8	86.1	87.91	1.87	1.94	1.91
x-Ray Omicron and Delta Variant	50	79.82	62.59	70.16	82.68	66.86	73.94	2.86	4.27	3.78
x-Ray Other Viral Pneumonia	60	78.19	69.9	73.81	80.06	72.29	75.98	1.87	2.39	2.17
x-Ray SARS	27	85.09	75.59	80.06	93.04	84.05	88.31	7.95	8.46	8.25
x-Ray Wildtype SARS-CoV-2	52	55.02	74.88	63.43	57.66	75.48	65.38	2.64	0.6	1.95

The table reports the number of samples (denoted as *n* in the header, indicating the sample size for each category), Precision, Recall, and F1 Score for each of the 11 pneumonia categories, along with the absolute improvement (in percentage points) introduced by LMF. Positive values in the improvement columns indicate performance gains, while negative values indicate slight performance trade-offs. The categories are ordered by sample size in descending order to highlight the inverse relationship between sample size and the magnitude of LMF-induced improvement: minority classes with fewer samples (e.g., x-Ray SARS, 27 samples) exhibit substantially larger gains, whereas majority classes (e.g., CT normal, 285 samples) show marginal decreases.

Table 5 summarizes the quantitative evaluation results of all nine models on the SARS-CoV-2 CT Scan Dataset, the second dataset in Section 2.1. LPC Transformer (Ours) achieved the best performance on all four metrics, with an accuracy of 94.57%, a recall of 94.56%, an accuracy of 94.58%, and an F1 score of 94.57%. HiFuse ranks second with an accuracy rate of 93.56%, a recall rate of 93.58%, an accuracy rate of 93.59%, and an F1 score of 93.56%. ResNet50 closely follows, becoming the third best performer with an accuracy rate of 92.96%, recall rate of 92.98%, accuracy rate of 93.00%, and F1 score of 92.96%. The accuracy of InternImage-B is 91.15%, the recall is 91.17%, the precision is 91.26%, and the F1 score is 91.14, which is slightly improved compared to its performance on the first dataset, possibly due to simplifying the binary classification task. ViT (accuracy 91.00%, recall 90.90%, accuracy 91.10%, F1 score 91.00%) and EfficientViT (accuracy 90.54%, recall 9,057%, accuracy 90.67%, F1 score 90.54%) occupy a moderate level, both exceeding the accuracy threshold of 90%. The accuracy of ConvNeXt V2 is 90.40%, and the values of all indicators are almost the same (recall 90.41%, accuracy 90.40%, F1 score 90.40%), indicating balanced performance but not outstanding. EfficientNet-b0 (accuracy 89.94%, recall 89.93%, precision 89.94%, F1 score 89.94%) and MobileNetV2 (accuracy 8,954%, recall 8,953%, accuracy 89.55%, F1 score 8,953%) occupy the last two positions, both below the accuracy threshold of 90%, demonstrating limited feature extraction capability in this task.

**Table 5:** Performance comparison of nine models on the SARS-CoV-2 CT-scan dataset (the second dataset).

Model/Metric	Accuracy (%)	Recall (%)	Precision (%)	F1 Score (%)
MobileNetv2_100	89.54	89.53	89.55	89.53
EfficientNet_b0	89.94	89.93	89.94	89.94
Vit_base_patch16_224	91.00	90.90	91.10	91.00
ConvNeXt V2	90.40	90.41	90.40	90.40
InternImage-B	91.15	91.17	91.26	91.14
EfficientViT	90.54	90.57	90.67	90.54
HiFuse	93.56	93.58	93.59	93.56
ResNet50	92.96	92.98	93	92.96
**Ours**	**94**.**57**	**94**.**56**	**94**.**58**	**94**.**57**

Performance comparison on the second dataset (SARS-CoV-2 CT-scan). Bold values indicate that our proposed method maintains superior performance and outperforms all other state-of-the-art models across all metrics.

It is worth noting that the model ranking comparison of the two data sets reveals a highly consistent pattern: although there are significant differences in task complexity, category granularity and data distribution, LPC Transformer maintains a leading position in the 11 categories of continuous learning data sets of COVID-19 and the binary SARS-CoV-2 CT Scan data set. The relative ranking of the remaining models remains relatively stable - HiFuse and ResNet50 consistently occupy the second and third layers, while lightweight models (MobileNetV2 and EfficientNet-b0) consistently rank at the bottom of both datasets. This strong consistency across datasets indicates that the observed performance hierarchy is attributed to the inherent architectural capabilities of the model, rather than dataset specific overfitting. More importantly, the ability of LPC Transformer to achieve the highest accuracy on datasets with different structures—with varying numbers of categories, different imaging sources, and different clinical backgrounds—provides convincing evidence that the proposed method effectively generalizes beyond specific distributions of training data. The dual advantages of its hybrid convolutional Transformer architecture in capturing local texture features and global context dependencies, coupled with the regularization effect of the hybrid loss function, enable the model to learn transferable and distinguishable representations that remain robust in heterogeneous data sources. These results collectively confirm that LPC Transformer is not only a dataset specific solution, but also a scalable framework with enormous potential for deployment in different clinical environments.

To comprehensively evaluate the computational efficiency and resource consumption of our model, we compare it with several representative backbones, including CNN-based (ConvNeXt-Base, ResNet50, EfficientNet-B0, MobileNetV2), Transformer-based (ViT-B/16, InternImage-B), and hybrid architectures (HiFuse-Tiny, EfficientViT-B2). As summarized in Table 6, our model contains 28.35 M parameters and requires 4.611 G FLOPs, which are substantially lower than those of ConvNeXt-Base (87.512 M, 15.354 G), InternImage-B (97.506 M, 16.175 G), and ViT-B/16 (86.498 M, 17.63 G), and are on par with ResNet50 (25.712 M, 4.132 G). In terms of peak GPU memory usage for a single image, our model consumes 420.08 MB, which is higher than ResNet50 (206.49 MB) and EfficientNet-B0 (109.17 MB), but notably lower than InternImage-B (542.53 MB). Regarding inference latency, our model achieves an average inference time of 10.178 ms, which is slower than lightweight CNNs (e.g., MobileNetV2: 4.434 ms, EfficientNet-B0: 5.572 ms) and HiFuse-Tiny (5.802 ms), yet remains competitive with ViT-B/16 (10.492 ms) while using only about one-third of its parameters and FLOPs.

**Table 6:** As shown in the table, we compare the parameter count, computational cost, peak GPU memory consumption, and average inference time across a diverse set of architectures, including convolutional networks (e.g., ConvNeXt-Base, ResNet50, EfficientNet-B0, MobileNetV2), Vision Transformers (e.g., ViT-B/16), and hybrid models (e.g., HiFuse-Tiny, EfficientViT-B2, InternImage-B).

Model	Params	FLOPs	Peak GPU Memory (single image)	Average Inference Time (single image)
ConvNeXt V2	87.512 M	15.354 G	331.44 MB	15.769 ms
HiFuse	82.714 M	8.145 G	223.91 MB	5.802 ms
InternImage-B	97.506 M	16.175 G	542.53 MB	15.016 ms
ResNet50	25.712 M	4.132 G	206.49 MB	6.231 ms
EfficientViT	24.674 M	1.609 G	290.76 MB	12.030 ms
Vit_base_patch16_224	86.498 M	17.63 G	222.13 MB	10.492 ms
EfficientNet_b0	5.29 M	0.385 G	109.17 MB	5.572 ms
MobileNetv2_100	3.526 M	0.306 G	92.81 MB	4.434 ms
**Ours**	28.35 M	4.611 G	420.08 MB	10.178 ms

The results provide a comprehensive overview of the efficiency trade-offs among different backbone families on a single-image basis.

Based on the above comparison, our model can be categorized as a Medium-scale architecture. Compared to heavy backbones such as ConvNeXt-Base, InternImage-B, and ViT-B/16, our model reduces the parameter count by approximately 67%–71% and FLOPs by 70%–74%, demonstrating a significant advantage in model compactness. However, when juxtaposed with extremely efficient models like MobileNetV2 and EfficientNet-B0, our model still exhibits a gap in memory footprint and inference speed, primarily due to the additional overhead introduced by our architectural design. Nevertheless, considering the substantial savings in parameters and FLOPs coupled with competitive inference efficiency against Transformer-based models, our model strikes a favorable balance between performance and computational cost, making it suitable for deployment in resource-constrained scenarios that can tolerate a moderate memory overhead.

To further compare the classification capabilities and feature space discriminative performance of various models, this study employs confusion matrices and t-SNE feature dimensionality reduction visualization methods to analyze the differences in representational ability among models from two dimensions: category recognition accuracy and feature clustering distribution. As shown in sub-figures A–F of Figures 6, 7.

**Figure 6:**
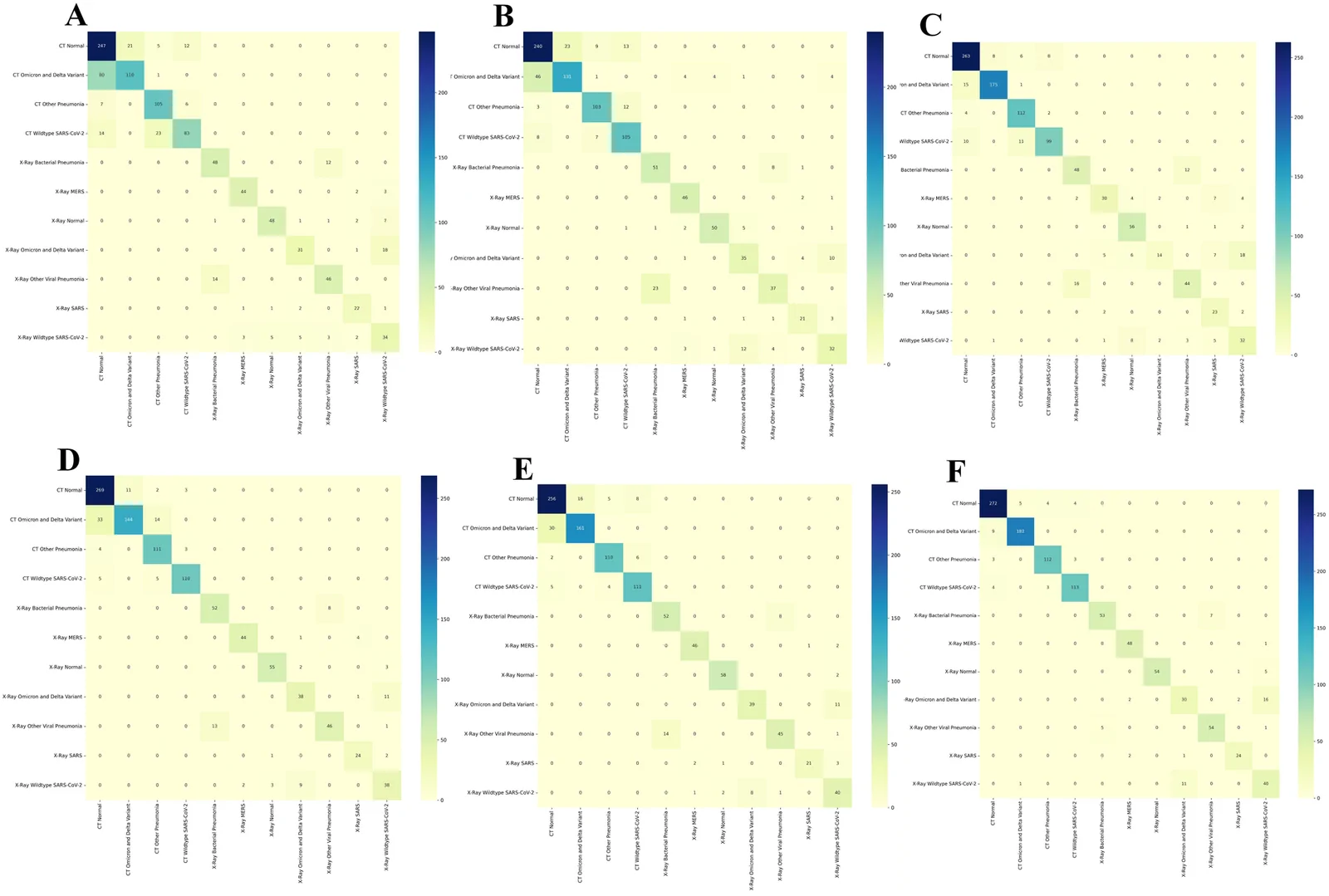
Confusion matrices for the six main models on the 11-class multimodal medical image classification task. **(A)** ConvNeXtV2; **(B)** Internimage-B model; **(C)** EfficientViT model; **(D)** Hifuse model; **(E)** ResNet50 model; **(F)** the proposed LPC-transformer model. The diagonal elements represent correctly classified samples, while off-diagonal elements indicate misclassifications between categories.

**Figure 7:**
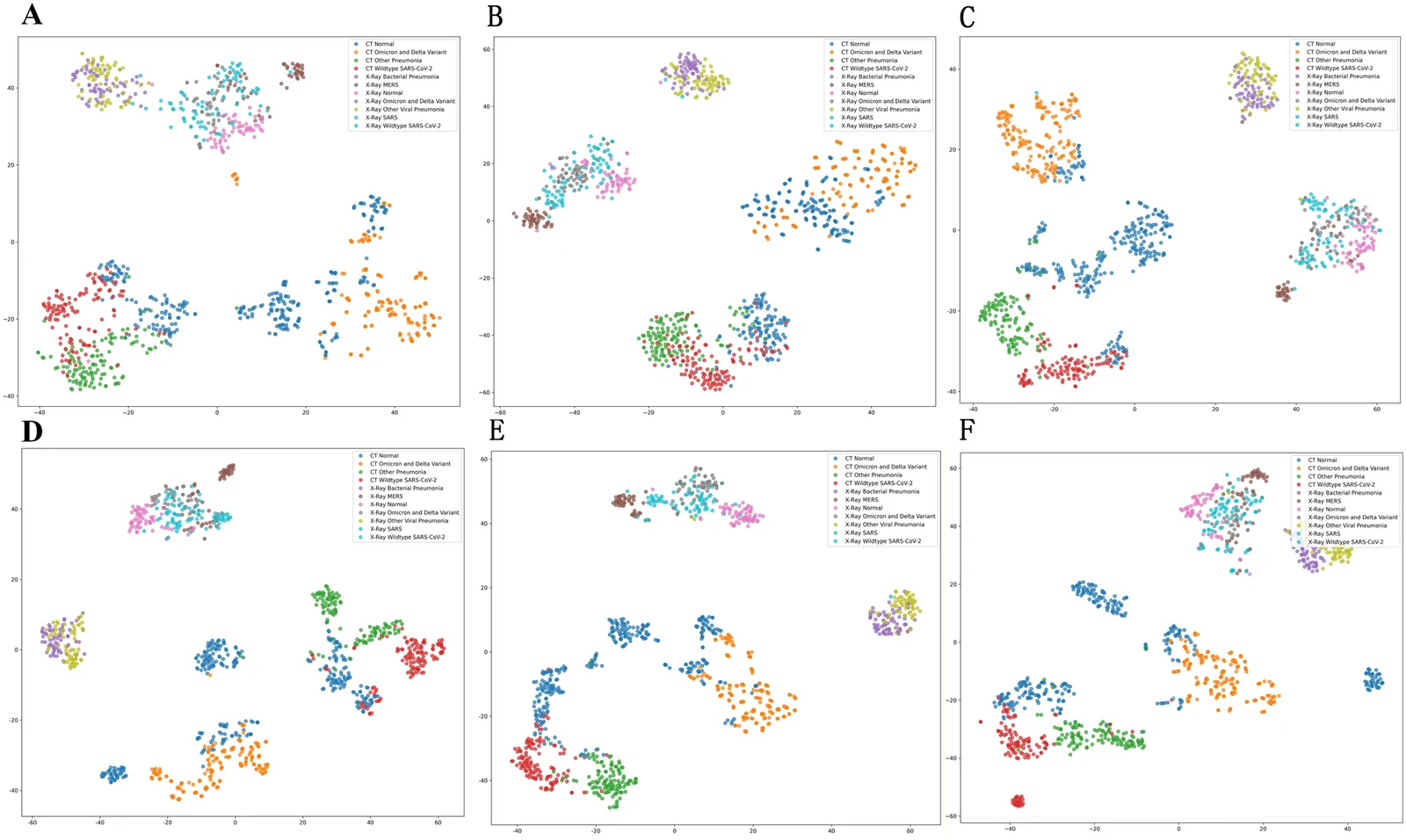
t-SNE 2D visualizations of feature representations for the six main models on the 11-class multimodal medical image classification task. **(A)** ConvNeXtV2; **(B)** Internimage-B model; **(C)** EfficientViT model; **(D)** Hifuse model; **(E)** ResNet50 model; **(F)** the proposed LPC-transformer model. Each point represents a sample, with colors indicating different categories. The tighter the intra-class clustering, the clearer the inter-class boundaries, and the less overlap between clusters, the stronger the model's discriminative capability.

The confusion matrix visually reflects the distribution of correct and incorrect classifications, as well as the misclassification patterns among the 11 types of medical image samples. The high diagonal element values and the scarcity of non-diagonal confusion entries indicate a high accuracy rate for single-class recognition and strong class differentiation ability. In the 11-class multimodal medical image classification task, the overall accuracy of LPC-Transformer reaches 86.38%, with a macro average F1 score of 84.87%. In the CT modality, its average class accuracy exceeds 90%, with only a very small number of cross-modal misclassified samples, indicating its high feature extraction ability for clear, high-contrast CT medical images. In the classification of low-resolution x-ray lung images with convergent lesion textures, a small amount of subtype confusion was observed, pointing to a clear direction for future optimization. Compared with other baseline models (A–E), LPC-Transformer (F) has the highest overall diagonal values in the confusion matrix, enabling accurate prediction of the vast majority of class samples with minimal cross-class misclassifications. Especially when distinguishing between lesion subtypes with highly similar pathological characteristics, as well as normal and mild cases, its misclassification rate is significantly lower than that of other comparative models, demonstrating the best overall consistency in class recognition.

t-SNE maps the high-dimensional features extracted by the model to a two-dimensional space. The tighter the clustering of samples within the same category, the greater the distance between clusters of different categories, and the less overlap between clusters, the stronger the classification and generalization ability of the model. As shown in Figure 6F, the LPC-Transformer model exhibits the highest degree of sample aggregation within each feature cluster, with clear cluster boundaries; the spacing between clusters of different categories is significantly larger, with almost no large-scale feature overlap. In contrast, the other comparative models all exhibit varying degrees of class cluster intermingling and modal feature mixing issues, indicating that the model has learned robust features with high inter-class discriminability, capable of capturing subtle differences between medical images of different categories.

To visually characterize each model's ability to extract and localize lesion features, this study employs Grad-CAM++ to generate visual heatmaps, as shown in Figure 8. By analyzing the feature response patterns of different network architectures, it is evident that there are significant differences in the models’ ability to focus on lesions. Transformer-based models such as InternImage-B, ConvNeXtV2, and EfficientViT generally suffer from attention diffusion issues, where activated regions tend to shift to irrelevant areas such as the mediastinum or normal lung tissue. These models exhibit varying degrees of false activation and feature redundancy, resulting in poor lesion matching. Although the multimodal fusion model Hifuse integrates features from multiple sources, it produces blurry depictions of lesion edges and has limited feature discrimination capabilities. In contrast, the traditional convolutional network ResNet50, leveraging its excellent local feature extraction capabilities, can stably focus on lesion regions, achieving high heatmap overlap and effective suppression of interference areas; its overall performance is second only to the model proposed in this paper. The heatmap generated by the proposed LPC-Transformer model features a compact activation range with clear boundaries; high-response regions show strong overlap with actual lesions, effectively suppressing spurious activations in the background and normal tissues. Visualization results demonstrate that this method possesses stronger lesion detection capabilities and fine-grained feature extraction, offering superior effectiveness and robustness in the diagnosis of thoracic diseases.

**Figure 8:**
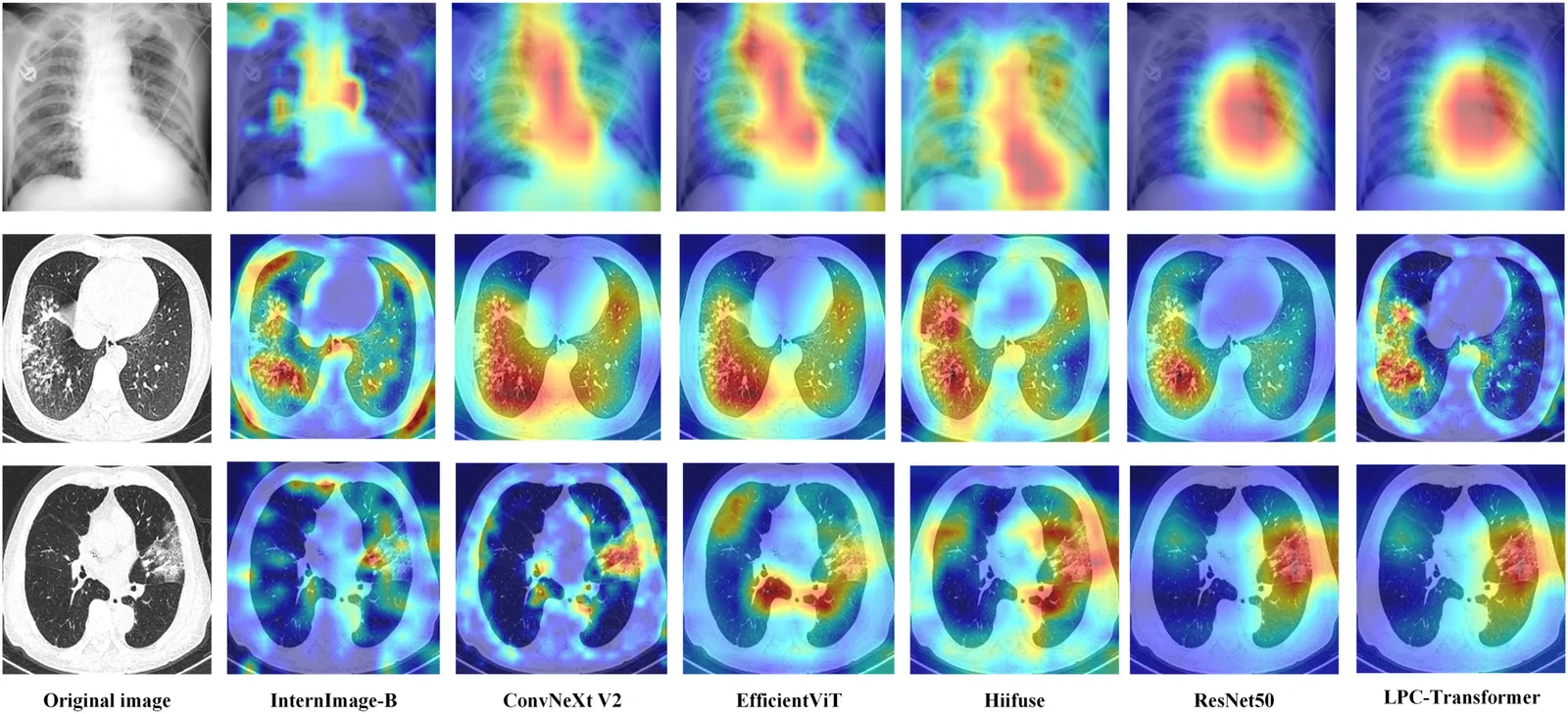
Grad-CAM attention heatmaps comparing feature localization across six models on three representative multimodal medical images. Rows (top to bottom): Chest x-ray, right-lung CT scan, and left-lung CT scan. Columns (left to right): Original image, InternImage-B, ConvNeXtV2, EfficientViT, Hifuse, ResNet50, and the proposed LPC-Transformer. Color gradient from blue to red indicates increasing attention weights, highlighting regions focused on by the model for classification.

## 4 Discussion

Pneumonia, a long-standing respiratory disease, has long posed a threat to human health. Since the 21st century, successive outbreaks of highly pathogenic coronaviruses (SARS-CoV, MERS-CoV, SARS-CoV-2, and their variants) have further highlighted the importance and urgency of differential diagnosis for pulmonary infectious diseases. Medical imaging modalities such as chest CT and x-rays are core tools for the differential diagnosis of pneumonia. Although different types of pneumonia exhibit certain characteristic imaging features—such as bacterial pneumonia typically presenting as unilateral focal consolidation, the original SARS-CoV-2 strain is characterized by bilateral peripheral ground-glass opacities, and the Omicron variant often presents as mild focal infiltrates—the imaging features of various types of pneumonia overlap significantly. In particular, both viral and bacterial pneumonia frequently exhibit pulmonary infiltrates, consolidation, or ground-glass opacities, posing a significant challenge to determining the underlying cause.

The aforementioned diagnostic difficulties stem from the complexity of pulmonary medical imaging, which manifests primarily in three aspects: first, individual variations and differences in symptom severity, leading to significant heterogeneity in imaging features even among patients with the same type of pneumonia, making standardized assessment difficult; second, performance variations in CT and x-ray equipment, which directly affect image quality and interfere with feature recognition; and third, the fusion effect between pulmonary images and the surrounding environment, which increases the difficulty of segmenting lesion boundaries and further impacts classification accuracy. This situation indicates that traditional imaging diagnostic methods struggle to meet the clinical demand for precise differentiation, creating an urgent need for efficient and accurate automated classification models to overcome these bottlenecks.

The LPC-Transformer model proposed in this study, based on the Vision Transformer architecture, effectively addresses the core challenges of feature extraction in pulmonary medical imaging. Its window mechanism precisely extracts local features from lung images and quantifies them, while the sliding window mechanism enables long-range feature learning. This decouples lung image regions from their surrounding environments, reducing environmental interference while strengthening the correlation between feature points in different lung regions, thereby efficiently extracting global features; Combined with optimizations such as the LMF hybrid loss function, the SCION optimizer, and the PolyReLU activation function, the model can better fit the data and converge to the optimal solution, while more efficiently learning complex feature representations of lung images. Experimental results demonstrate that, in the multiclass pneumonia image classification task, LPC-Transformer outperforms SwinT across all evaluation metrics—accuracy, recall, precision, and F1-score—with improvements ranging from 2% to 4%. This fully demonstrates the model's superiority in pneumonia image classification, indicating strong potential for clinical application and providing reliable technical support for the precise differential diagnosis of pneumonia.

This study still has certain limitations that require further refinement in future research. First, the training data for the LPC-Transformer model is primarily limited to two imaging modalities—CT and x-ray—and its applicability to other lung imaging modalities (such as MRI) has not yet been validated; the model's robustness and stability still require further evaluation. Second, as the model is based on the Swin-Transformer backbone and integrates multiple optimization strategies, it has high computational resource requirements for both training and inference, making it difficult to adapt to clinical settings with limited resources. These limitations also point the way for future research: on the one hand, the imaging modalities of the training data can be expanded to explore the application of multimodal fusion techniques in pneumonia classification, thereby further enhancing the model's generalization ability; on the other hand, efforts can be made to design a lightweight network by streamlining parameters and optimizing the architecture to reduce computational requirements, achieving a balance between classification performance and operational efficiency, and facilitating the model's translation into practical clinical applications.

Furthermore, in light of current clinical needs for pneumonia prevention and control, the practical value of the LPC-Transformer model lies not only in improving the accuracy of pneumonia type identification but also in providing technical support for rapid clinical diagnosis and timely intervention. This is particularly relevant for pneumonia caused by SARS-CoV-2 variants, as the model can quickly distinguish the imaging features of infections caused by different variants, thereby informing the development of treatment strategies. In the future, as research progresses, the model's clinical adaptability can be further optimized by integrating clinical case data, driving the large-scale application of automated image classification technology in the diagnosis of pulmonary infectious diseases and supporting global pneumonia prevention and control efforts.

## 5 Conclusions

Addressing the challenges of classifying pulmonary medical images and the insufficient accuracy of traditional methods, this study proposes the LPC-Transformer model for the automatic classification of multi-category pneumonia. The main conclusions are as follows: The complexity of pulmonary medical images (individual variability, device variability, and difficulty in boundary segmentation) and the overlap in imaging features among different types of pneumonia are the core reasons for the difficulty in accurately differentiating pneumonia, and traditional imaging diagnostic methods struggle to meet clinical needs. Leveraging a multi-module architecture and various optimization strategies, this model possesses strong capabilities for extracting complex image features. Comparative experimental results show that, compared to the baseline model SwinT, this model achieves performance improvements ranging from 2% to 4% across all evaluation metrics, demonstrating consistent and notable comprehensive performance advantages and providing a viable technical solution for the accurate clinical diagnosis of pneumonia. This study has certain limitations. The applicability and robustness of the LPC-Transformer model across multiple imaging modalities remain to be validated, and it is highly dependent on computational resources. In the future, model performance can be further optimized by expanding imaging modalities and implementing network lightweight design, thereby facilitating its translation into clinical practice and providing stronger support for the prevention, control, and diagnosis of pulmonary infectious diseases.

## Data Availability

Publicly available datasets were analyzed in this study. This data can be found here: https://www.kaggle.com/datasets/mustai/continual-learning-of-covid19/data.
